# Extending Goldberg’s method to parametrize and control the geometry of Goldberg polyhedra

**DOI:** 10.1098/rsos.220675

**Published:** 2022-08-10

**Authors:** Yuanpeng Liu, Ting-Uei Lee, Anooshe Rezaee Javan, Yi Min Xie

**Affiliations:** Centre for Innovative Structures and Materials, School of Engineering, RMIT University, Melbourne 3001, Australia

**Keywords:** Goldberg polyhedra, geometry, planarity, spherical polyhedra, parametrization, optimization

## Abstract

Goldberg polyhedra have been widely studied across multiple fields, as their distinctive pattern can lead to many useful applications. Their topology can be determined using Goldberg’s method through generating topologically equivalent structures, named cages. However, the geometry of Goldberg polyhedra remains underexplored. This study extends Goldberg’s framework to a new method that can systematically determine the topology and effectively control the geometry of Goldberg polyhedra based on the initial shapes of cages. In detail, we first parametrize the cage’s geometry under specified topology and polyhedral symmetry; then, we manipulate the predefined independent variables through optimization to achieve the user-defined geometric properties. The benchmark problem of finding equilateral Goldberg polyhedra is solved to demonstrate the effectiveness of the proposed method. Using this method, we have successfully achieved nearly exact spherical Goldberg polyhedra, with all vertices on a sphere and all faces being planar under extremely low numerical errors. Such results serve as strong numerical evidence for the existence of this new type of Goldberg polyhedra. Furthermore, we iteratively perform *k*-means clustering and optimization to significantly reduce the number of different edge lengths to benefit the cost reduction for architectural and engineering applications.

## 1. Introduction

### 1.1. Background

Goldberg polyhedra are three-dimensional structures made up of planar hexagons and pentagons with exactly three faces that meet at each vertex [1]. Uniquely, a Goldberg polyhedron possesses a certain type of polyhedral (icosahedral, tetrahedral or octahedral) symmetry [2], meaning the same faces may be repeated periodically through transformations of reflection and rotation. Due to the distinctive pattern, Goldberg polyhedra have demonstrated great significance in various fields. Besides the prominent designs of geodesic domes in architecture [3,4], they have frequently appeared in artistic and architectural applications [5–7]. Moreover, they can resemble the form of virus capsids [2,8] and fullerenes [9,10], hence further gaining intensive attention from biology and chemistry.

A Goldberg polyhedron is uniquely determined by its topology and geometry. The topology [11] describes the connectivity between vertices, edges and faces, whereas the geometry portrays the elements’ shape such as length, angle and area. The topology can be derived using Goldberg’s method [1], which will be explained in detail in §1.2, or similar approaches [2,3,12] through constructing topologically equivalent polyhedral/spherical *cages*. The generated cages are three-dimensional structures geometrically analogous to Goldberg polyhedra, which possess the same type of polyhedral symmetry; however, their faces are typically not planar, thereby these structures are named ‘cages’ to distinguish with standard ‘polyhedra’. With a fixed topology, the cage’s geometry is fully determined by the positions of its vertices. Unfortunately, none of these methods has further attempted to alter the positions of vertices to achieve cages with planar faces (i.e. Goldberg polyhedra) or other geometric properties.

Specific geometric properties are of great significance in real-life applications. For example, in architecture and engineering [13–15], faces being planar (planarity) is demanded for representing surfaces built with flat glass/metal panels [16]; all edges being of equal length (equilaterality) enables mass production of steel/wood struts to reduce the unit cost [17–19]; all vertices lying on a sphere (inscribability) may be required by spherical buildings [3]. Designs with the desired geometric properties could bring remarkable benefits for fabrication and construction.

There are various methods to generate cages with different geometric properties. For example, one can derive spherical cages by vertex elimination [5], cages with planar faces through three-dimensional reciprocal construction [5], cages with equal-length edges by trigonometry [20] or optimization [21], and ‘the roundest polyhedra’ using numerical approaches [7,22,23]. Remarkably, cages with planar faces and simultaneously equal-length edges can be obtained by nulling the dihedral angle discrepancies [21]. However, one special class of cages, with all vertices on a sphere and all faces being planar, have not been found or proved non-existent by previous studies.

### 1.2. Goldberg’s method

#### 1.2.1. Main steps

**Figure 1.**
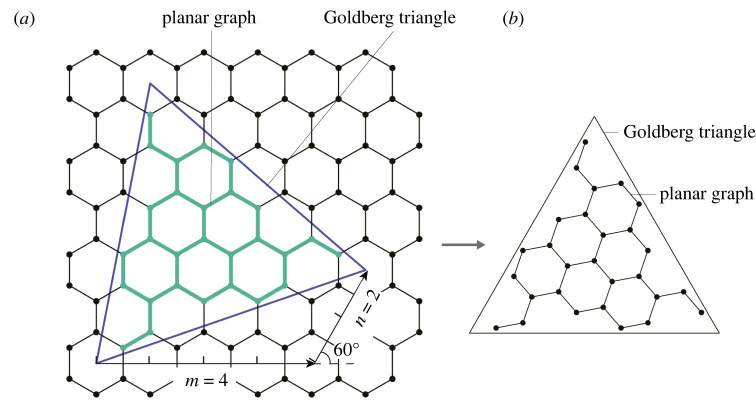
Construction of the planar graph. (*a*) An equilateral triangle, the Goldberg triangle, is constructed in a hexagonal grid, with its bottom edge spanning *m* = 4 hexagons in the horizontal direction and *n* = 2 hexagons at 60°. (*b*) The planar graph, constituted by the vertices and edges inside the Goldberg triangle, is extracted.

**Figure 2.**
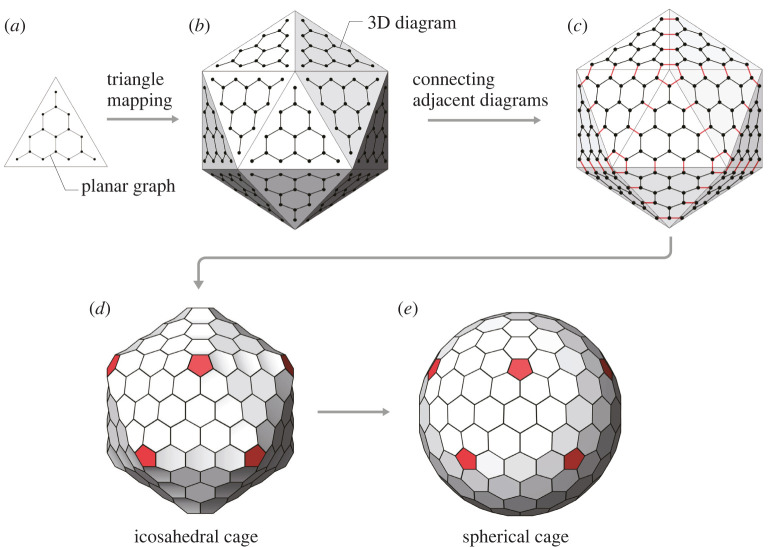
Construction of the icosahedral/spherical cage based on the planar graph. (*a*) The planar graph together with the Goldberg triangle. (*b*) The planar graph is transformed on to the faces of an icosahedron through triangle mapping, leading to an identical three-dimensional diagram on each face. (*c*) The three-dimensional diagrams of adjacent faces are connected with additional edges (in red), generating an untied and closed geometry. (*d*) The obtained icosahedral cage with 12 pentagonal faces in red. (*e*) The vertices of the icosahedral cage are projected to a sphere, forming a spherical cage.

Goldberg’s method [1] is a framework for constructing polyhedral/spherical cages with prescribed topology and certain types of polyhedral (icosahedral, tetrahedral and octahedral) symmetry. It consists of two main steps, as shown in figures 1 and 2, respectively. Here, we take the case of icosahedral symmetry as an example to illustrate the framework. The construction of cages with the other types of symmetry follows the same processes.

The first step determines the planar graph in the following way. In the beginning, a grid made up of regular hexagons is established in the two-dimensional plane. Over the grid, one draws an equilateral triangle, named the *Goldberg triangle*, according to a prescribed non-negative integer pair (*m*, *n*). Starting from a hexagon’s centre, one first takes *m* steps of hexagons rightward and then turns 60° anticlockwise with another *n* steps (figure 1*a*) to the end. The line connecting the start and end positions is the Goldberg triangle’s bottom edge, with which the entire equilateral triangle can be easily created. Then, the planar graph, constituted by the vertices and edges inside the Goldberg triangle, can be extracted (figure 1*b*); notably, the number of vertices equals *T* = *m*^2^ + *mn* + *n*^2^.

The second step employs the planar graph to generate polyhedral/spherical cages. First, the planar graph (figure 2*a*) is transformed on to an icosahedron by triangle mapping, leading to an identical three-dimensional diagram on each face (figure 2*b*). Then, three-dimensional diagrams of adjacent faces are connected with additional edges (figure 2*c*), creating a united and closed three-dimensional geometry. The resulted geometry is an icosahedral cage (figure 2*d*), whose vertices may be further projected on to a sphere, forming a spherical variant (figure 2*e*). It should be highlighted that the cage’s topology is entirely determined by (*m*, *n*); thereby, we use C(*m*, *n*) and GP(*m*, *n*) to denote the cages and Goldberg polyhedra with the specific topology, respectively. Notably, C(*m*, *n*) and GP(*m*, *n*) both contain 20*T* vertices, 30*T* edges, 9*T* hexagonal faces and 12 pentagonal faces [5]; however, at this point, the cage’s hexagonal faces are typically not planar.

#### 1.2.2. Merits and limitations

Goldberg’s method can systematically generate a series of cages with a specific topology corresponding to (*m*, *n*) values. Besides, the cages are in a highly structured form of icosahedral symmetry, which will be discussed in detail in §2.1. However, the main limitation is that Goldberg’s method lacks control over the cages’ geometry. For instance, cages with planar faces or other desired geometric properties may not be effectively generated using this method.

### 1.3. Contributions and the outline of this study

This study presents a new method to construct cages with complete control over the geometry by extending Goldberg’s framework [1]. The method contains two steps. First, the cage’s geometry is parametrized under specified topology and icosahedral symmetry constraints. Second, the parametrized geometry is controlled by manipulating the predefined independent variables through extra optimization techniques. Using this method, cages with specific geometric properties can be explored, such as equilateral Goldberg polyhedra (i.e. cages with equal-length edges and planar faces) [21]. Particularly, we have achieved nearly exact spherical Goldberg polyhedra, with all vertices on a sphere and all faces being planar under extremely low numerical errors. The obtained results offer strong numerical evidence for the existence of such spherical Goldberg polyhedra which have not been found previously.

This paper is outlined as follows. In §2, we elaborate on the parametrization of the cage’s geometry based on Goldberg’s method. Section 3 describes the formulation of specific geometric constraints and explains the optimization method. Section 4 discusses the construction of equilateral Goldberg polyhedra using the proposed framework. Section 5 presents a numerical investigation on spherical Goldberg polyhedra, followed by a conclusion in §6.

## 2. Parametrizing the cage’s geometry

### 2.1. The icosahedral symmetry

**Figure 3.**
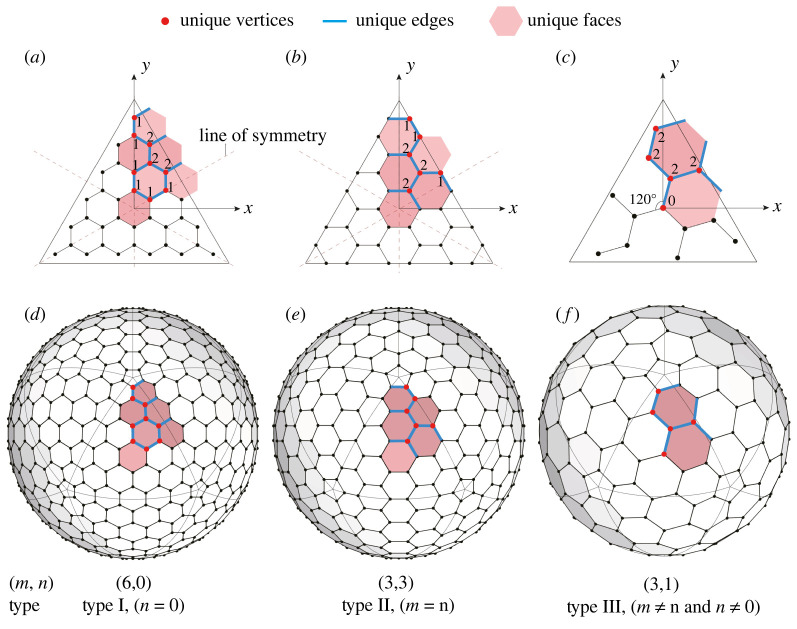
Labellings of the unique vertices, edges and faces in the planar graphs (*a*–*c*) and spherical cages (*d*–*f*) of different types. In (*a*–*c*), the numbers represent the degrees of freedom of the corresponding unique vertex.

The icosahedral symmetry is a critical constraint for the parametrization process. As a special geometric property, it contains two main characteristics. First, the 20 three-dimensional diagrams on the icosahedron’s faces are congruent (figure 2*b*), meaning they are exactly the same and can be transformed into each other through rigid motions. This is obvious as the planar graph is mapped on to 20 identical equilateral triangles. Second, the planar graph itself also contains certain degrees of symmetry. Such symmetry is related to the cages’ type, which is defined as: type I for *n* = 0, type II for *m* = *n* and type III for *n* ≠ *m* and *n* ≠ 0. Then, for types I and II, the planar graph contains reflection symmetry, with the Goldberg triangle’s medians as lines of symmetry, as shown in figure 3*a*,*b*. Incongruously, for type III, the planar graph possesses rotational symmetry of order 3, where the shape is invariant under a rotation of 120° (figure 3*c*). The planar graph’s symmetry is resulted from the interaction between the Goldberg triangle and the hexagonal grid.

Based on the understanding of icosahedral symmetry, the *unique elements* can then be determined. The unique elements are the smallest group of vertices, edges and faces needed to recover the entire cage through duplication and rigid transformation. By ruling out the repeated ones according to symmetry, the unique vertices, unique edges and unique faces can be identified in the planar graph as well as the three-dimensional cage, as shown in figure 3. Note that the 12 pentagonal faces always remain equilateral and planar, thereby considered separately.

It should be noted that cages may possess other possible types of symmetry. For example, during the triangle mapping, the planar graph can be mapped on to the triangular faces of a tetrahedron or an octahedron, forming cages with tetrahedral or octahedral symmetry. Furthermore, the planar graph can also be extracted from a quad grid using a square instead of the Goldberg triangle and then mapped on to the quadrilateral faces of a cubic, forming cages with hexahedral symmetry. However, despite the variation in the symmetry of cages, the unique elements can be analysed and determined in a similar way. In this study, we only take the case of icosahedral symmetry as an example to demonstrate the proposed framework.

### 2.2. Defining the independent variables

#### 2.2.1. In the two-dimensional planar graph

The degrees of freedom in the planar graph are closely related to the positions of unique vertices. In a two-dimensional Cartesian coordinate system, each unique vertex typically contains two degrees of freedom, the *x*- and *y*-coordinate. However, to maintain the cage’s topology and symmetry, additional constraints exist. For instance, the unique vertices on the Goldberg triangle’s boundary or the lines of symmetry should be restricted to those lines. Figure 3*a*–*c* shows three examples with each unique vertex labelled with the corresponding degrees of freedom in the planar graph.

With all degrees of freedom determined, we use an independent variable to represent each of them. In doing so, all vertices’ two-dimensional coordinates in the planar graph are described explicitly in terms of a list of independent variables, denoted as *C*. In order to be consistent with the following three-dimensional operations, all two-dimensional vertices in the same plane are pre-converted into three-dimensional formats by adding a *z*-coordinate for each vertex. Specifically, the value of the *z*-coordinate must be the same for each vertex, and must be non-zero to avoid singularity that may lead to an error in the following matrix calculation.

#### 2.2.2. In the three-dimensional space

The vertices in the planar graph are then transformed on to an icosahedron’s faces through triangle mapping. In this study, the mapping is a combination of uniform scaling and rigid transformation, which pastes the scaled planar graph to each face of the icosahedron. In analytical geometry, this process can be represented by a transformation matrix *F*, linking the vertices’ coordinates before and after mapping using equation (2.1).

2.1 $$V_{i}=FV_{p},$$

where *V*_*p*_ and *V*_*i*_ contain the coordinates of vertices in the planar graph and on the icosahedron, respectively. *F* can be derived through the spacial relationship between the Goldberg triangle and the icosahedron’s triangles as

2.2 $$F=V_{ti}(V_{tp}^{-1}E),$$

where *E* is the identity matrix, *V*_*tp*_ and *V*_*ti*_ contain the coordinates of the Goldberg triangle’s vertices and the icosahedron’s triangles, respectively. In equation (2.1), *V*_*p*_ is in terms of *C*, whereas *F* is a constant matrix; thereby, the generated *V*_*i*_ is only related to *C*, indicating that no additional independent variable has been introduced during the triangle mapping. It is also worth mentioning that equations (2.1) and (2.2) would still apply if the icosahedron is replaced with a tetrahedron or octahedron.

The vertices on the icosahedron can further drift away from the surface in the radial direction. This process is equivalent to projecting a vertex from the icosahedron to a sphere of the same centre. For each unique vertex, its distance to the sphere centre, which is the corresponding radius, can be of any value and is independent from those of other unique vertices. Therefore, we introduce one additional independent variable for each unique vertex to represent the radius and denote the list of radii as *R*. Knowing the radius, calculating the vertices’ coordinates after projection would be a simple task. Eventually, all vertices’ coordinates can be represented by the *C* and *R*, with no more degrees of freedom. In other words, the cage’s geometry has already been parametrized in terms of these independent variables.

## 3. Optimizing the cage’s geometry

### 3.1. Formulating geometric properties

In order to achieve specific properties, one should pre-describe these properties as equations of the independent variables. In this study, three geometric properties, planarity, equilaterality and inscribability, are exemplarily formulated in terms of *C* and *R*. Note that, due to the icosahedral symmetry, these geometric constraints only need to be applied to the unique elements.

Firstly, planarity can be achieved by flattening all unique faces. For the *i*th unique face, we begin by denoting its vertices as *V*_1*i*_, *V*_2*i*_, *V*_3*i*_, *V*_4*i*_, *V*_5*i*_ and *V*_6*i*_, of which the sequence can be random. Then, any three vertices, e.g. *V*_1*i*_, *V*_2*i*_ and *V*_3*i*_, are chosen to determine a base plane, meanwhile requiring the other three vertices to stay on it. More specifically, the distances from *V*_4*i*_, *V*_5*i*_ and *V*_6*i*_ to the base plane, *d*_4*i*_, *d*_5*i*_ and *d*_6*i*_, must equal zero, leading to three constraint equations (equation (3.1)).

3.1 $$\begin{array}{ll} & d_{4i}(C,R)=0,\\ & d_{5i}(C,R)=0\\ \mathrm{and}\; & d_{6i}(C,R)=0. \end{array}\}$$

Secondly, equilaterality requires all unique edges’ lengths to be equal. We denote the *j*th unique edge’s length as *L*_*j*_, where 0 < *j* < *a*, and *a* is the number of unique edges. Then, this property can be described as equation (3.2).

3.2 $$L_{j}(C,R)-L_{\;j+1}(C,R)=0.$$

Thirdly, inscribability demands all unique vertices to be on the same sphere. Therefore, all unique vertices’ radii must be equal, as shown in equation (3.3), where *R*_*k*_ refers to the *k*th unique vertex’s radius, and 0 < *k* < *b*, with *b* being the number of unique vertices.

3.3 $$R_{k}(C,R)-R_{k+1}(C,R)=0.$$

Other user-defined geometric properties are also compatible. In general, any geometric property is linked to vertices’ coordinates, thereby can be formulated as a geometry function *f* of the independent variables in the form of equation (3.4).

3.4 f (C,R)=0.

Eventually, a system of equations can be established by formulating and combining the desired geometric properties. It is worth noting that our method can be easily adapted to various scenarios with different geometric targets simply by replacing, adding or removing the corresponding equations in the system. With the system specified, we can try to achieve the desired geometric properties by seeking the solution to these equations.

### 3.2. Optimization method

A system of equations can be solved either analytically or numerically. Ideally, one would expect the analytical solution due to its exactness. However, solving multiple equations analytically could be extremely difficult, especially when the equations are mostly nonlinear. Therefore, in this study, we adopt a more generic approach, the numerical method (optimization), to solve the problem but put harsh conditions on the geometric errors to pursue the *nearly exact solutions*.

#### 3.2.1. Optimization objective

This study solves the nonlinear equations using the *fsolve* function [24,25] in Matlab [26]. The core of *fsolve* is the Levenberg–Marquardt algorithm (LMA) [27], which finds the solutions in the sense of least squares. More specifically, LMA converts the original root-finding problem to an unconstrained optimization problem, as shown in equation (3.5).

3.5 $$\min\;F=\sum_{\;p=1}^{q}w_{p}f_{p}^{2}(C,R),$$

where *F* is the objective function, *q* is the total number of equations and *w*_*p*_ = 1 is the weight of the *p*th geometry function *f*_*p*_. Starting from an initial point, LMA iteratively adjusts *C* and *R* based on the gradient information to reduce *F*. Ideally, *F* will be minimized to exactly zero, where all geometry functions are nulled, meaning all geometric properties are perfectly realized. Unfortunately, this is in general not achievable with a numerical method; nevertheless, we can still improve the results’ quality by avoiding the *spurious solutions* and pursuing the *nearly exact solutions*.

#### 3.2.2. Spurious solution

LMA may stop when *F* has not reached zero yet, producing spurious solutions. Specifically, ‘spurious’ means the geometric constraints are not really satisfied, and inequalities still exist between the two sides of equations. Typically, spurious solutions may appear due to the following two reasons.

First, the number of geometric constraints is excessive; thus, there exists no valid solution at all. In this case, LMA will try to satisfy all geometric constraints to the most extent and return a least-square solution, with *F* remaining a relatively small number (e.g. 10^−5^). In order to further reduce *F*, the geometric constraints need to be relaxed.

Second, even given the existence of a valid solution, LMA may still fail to find it due to the algorithm’s inherent deficiency. Like other gradient-based methods, LMA may be stuck at a local minimum, especially when the objective function is highly nonlinear and the initialization is very poor, i.e. far away from the solution. Nevertheless, a good initial guess could help to improve the quality of results. According to our experiences, the spherical cage’s configuration (figure 2*e*) in Goldberg’s method may be a good initialization, thereby being adopted for the numerical tests in §4–5.

#### 3.2.3. Nearly exact solutions

Generally, *F* cannot reach exactly zero but may remain an extremely small value, e.g. 10^−30^. This is due to the interference of rounding errors [22], which result from the inexactness in the representation of real numbers and the arithmetic operations done with them. For example, the theoretical value of ln(1) is 0 obviously, yet it equals 2.2204 × 10^−16^ in Matlab, due to the limitation of 16 digits of precision [20]. In general, the rounding error commonly exists in algorithms implemented by the machinery, and typically is acceptable for real-life projects.

Considering the rounding errors, we try to achieve ‘nearly exact solutions’ by putting harsh conditions on the geometric errors. In this study, three geometric errors, *σ*_*p*_, *σ*_*e*_ and *σ*_*i*_, are introduced regarding planarity, equilaterality and inscribability, respectively. Specifically, *σ*_*p*_ denotes the maximum gap from a vertex to the corresponding base plane, *σ*_*e*_ shows the maximum deviation from an edge length to the average value, and *σ*_*i*_ represents the maximum distance from a vertex to the target sphere. Then, we assume that, if the related errors are all below 10^−15^, the objective function will be ‘close enough’ to 0, thereby the analytical solutions exist, and can be approximated by the obtained numerical solutions, namely ‘nearly exact solutions’.

## 4. Equilateral Goldberg polyhedra

Equilateral Goldberg polyhedra are cages composed of planar faces and equal-length edges. They were first discovered by Schein & Gayed [21] through solving equations with respect to internal angles. Equilateral Goldberg polyhedra are very rare, as there may exist only one unique solution for a given topology [21]. In architecture and engineering, equilateral Goldberg polyhedra are of great significance in reducing the construction cost, as merely one type of edge is required, and all faces are planar.

**Figure 4.**
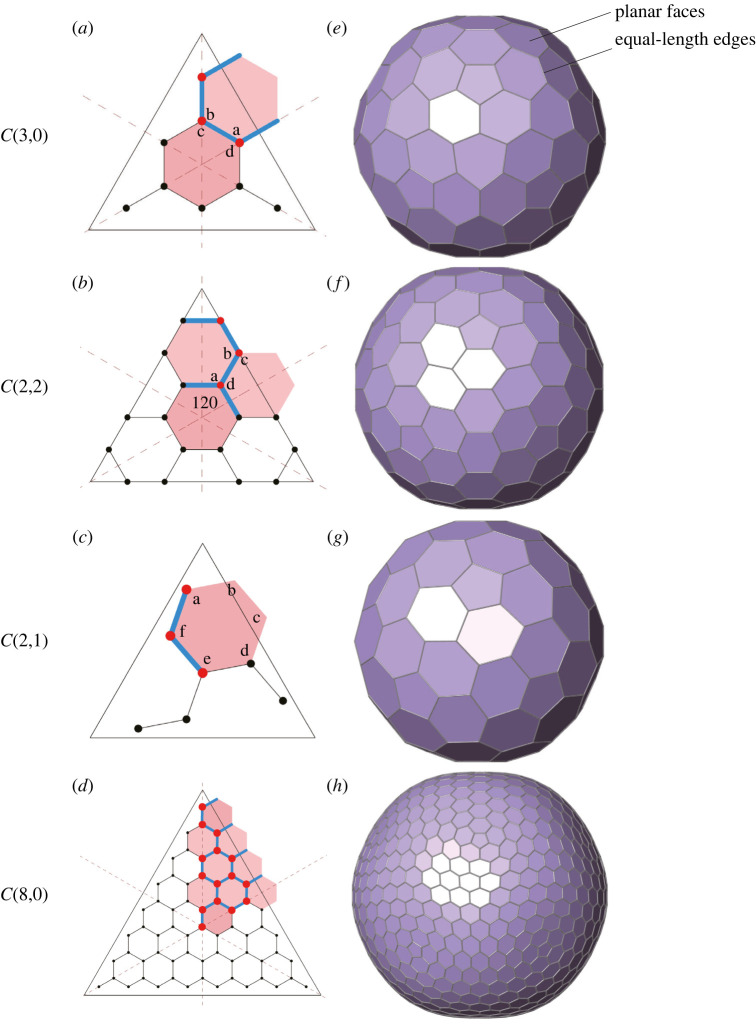
Equilateral and planar cages C(3,0), C(2,2) and C(2,1), and their corresponding planar graphs.

To examine the effectiveness of the proposed framework, we aim to solve the benchmark problem of finding equilateral Goldberg polyhedra. We exemplarily test four different cages, C(3,0), C(2,2), C(2,1) and C(8,0), by formulating and solving the equations regarding planarity and equilaterality. The generated geometries are shown in figure 4*e*,*f*, with corresponding data detailed in table 1, cases 1–4, respectively.

**Table 1.** Summarized data for all numerical examples in this study. In properties, ‘E’ denotes ‘equilateral’, ‘P’ denotes ‘planar’ and ‘S’ denotes ‘spherical’. For spherical cages, all vertices are constrained to be on a unit sphere. For cases 1–4, the range of radius *r* is listed in *σ*_*i*_. For cases 8–10, *σ*_*e*_ denotes the maximum difference between the pentagon’s edge length *l*_*p*_ with 0.100. For cases 11–13, *σ*_*e*_ denotes the maximum deviation between each edge length and its corresponding cluster centre.

case	(*m*, *n*)	properties	*σ*_*p*_	*σ*_*e*_	*σ*_*i*_	runtime (s)
1	(3,0)	E & P	0.48 × 10^−16^	0.28 × 10^−16^	\ (*r*: 0.971-1)	0.200
2	(2,2)	E & P	0.82 × 10^−16^	0.28 × 10^−16^	\ (*r*: 0.974-1)	0.253
3	(2,1)	E & P	0.16 × 10^−16^	0.56 × 10^−16^	\ (*r*: 0.975-1)	0.260
4	(8,0)	E & P	0.84 × 10^−16^	0.87 × 10^−16^	\ (*r*: 0.963-1)	0.562
5	(3,0)	S & P	0.29 × 10^−16^	\	0	0.255
6	(2,2)	S & P	0.41 × 10^−16^	\	0	0.378
7	(2,1)	S & P	0.39 × 10^−16^	\	0	0.278
8	(3,0)	S & P & *l*_*p*_ = 0.100	0.29 × 10^−16^	0.14 × 10^−16^	0	0.290
9	(2,2)	S & P & *l*_*p*_ = 0.100	0.54 × 10^−16^	0	0	0.322
10	(2,1)	S & P & *l*_*p*_ = 0.100	0.53 × 10^−16^	0	0	0.323
11	(8,0)	S & P & *k* = 20	1.21 × 10^−16^	0	1.11 × 10^−16^	0.706
12	(8,0)	S & P & *k* = 15	1.31 × 10^−16^	0.28 × 10^−16^	1.11 × 10^−16^	23.319
13	(8,0)	S & P & *k* = 4	2.31 × 10^−5^	9.98 × 10^−5^	8.81 × 10^−5^	56.941

It is shown in table 1 that the planarity and equilaterality errors for cases 1–4 are extremely low, all below 10^−15^. This indicates that the solutions are nearly exact. To further validate the accuracy of the results, we calculate the corresponding internal angles to compare with the results of Schein & Gayed [21]. Note that, for C(8,0), no corresponding result is available in [21]; hence, it is not included in the comparison. The comparison results are presented in table 2, which clearly shows that the values of internal angles match perfectly. The numbers remain the same up to the 10th decimal place, and the difference from the 11th decimal place (in bold) is mostly due to the different data precision. In summary, such results suggest that our method can achieve nearly exact equilateral Goldberg polyhedra with extremely low errors.

**Table 2.** The comparison of internal angles between data of [21] and our results for equilateral Goldberg polyhedra. The angle IDs correspond to the labellings in figure 4. The numbers up to the 10th decimal places are the same, and become different from the 11th decimal places, which are in bold.

(*m*, *n*)	type	angle ID	Schein & Gayed [21]	our method
(3,0)	I	a	124.8940990970**6**	124.8940990970**552**
		b	110.2118018058**9**	110.2118018058**897**
		c	104.4775121859**3**	104.4775121859**299**
		d	135.5224878140**7**	135.5224878140**701**
(2,2)	II	120	120.0000000000**0**	120.0000000000**000**
		a	125.2643896827**6**	125.2643896827**546**
		b	109.4712206344**9**	109.4712206344**907**
		c	138.1896851042**2**	138.1896851042**214**
		d	110.9051574478**9**	110.9051574478**893**
(2,1)	III	a	124.2065953282**7**	124.2065953282**647**
		b	124.2065953282**7**	124.2065953282**647**
		c	108.2542976449**7**	108.2542976449**717**
		d	131.1524094593**2**	131.1524094593**187**
		e	117.5207572132**2**	117.5207572132**227**
		f	114.6593450259**6**	114.6593450259**577**

The required runtime for all cases is listed in table 1. Specifically, runtime denotes the overall time consumed by the optimization process to find the solutions, not including the time for pre-processing (formulating the equations) and post-processing (reconstructing the model of the entire cage). For cases 1–4, the runtime is very low, all less than 0.6 s, even for the most complex cage C(8,0) with 1280 vertices, 1920 edges and 642 faces. The prompt response of our framework is mainly attributed to the exploitation of the icosahedral symmetry, as this process can significantly reduce the number of free variables and constraints. For example, as shown in figure 4*d*, for C(8,0), only 15 unique vertices, 20 unique edges and 9 unique faces are involved in the optimization problem, which are just 1.17%, 1.04% and 1.40% of all vertices, edges and faces, respectively.

## 5. Spherical Goldberg polyhedra

Spherical Goldberg polyhedra are cages with all vertices on a sphere and all faces being planar. Such unusual polyhedra have not been found previously; their geometries remain unknown. As stated in [5]: *Method 1 attempts to give an inscribed result, with vertices on the sphere, but that is not generally attainable with planar faces. The dodecahedron and truncated icosahedron are inscribable, but whether or how the more complex GPs are inscribable while preserving symmetry is an open problem.* Compared with equilateral Goldberg polyhedra, spherical Goldberg polyhedra can better approximate a sphere, hence may be more desirable for architectural designs of spherical buildings. This study approaches spherical Goldberg polyhedra from the aspect of numerical modelling. We present three cases with different numbers of additional constraints imposed on spherical Goldberg polyhedra to investigate their existence, uniqueness and remaining degrees of freedom.

### 5.1. Case I: with no additional constraint

The existence of spherical Goldberg polyhedra is investigated using the proposed framework. Specifically, given cages with specific topology, we formulate and solve the equations regarding planarity and inscribability. If the resulted errors are all below the specified threshold (10^−15^), the obtained geometries are nearly exact, which provide numerical evidence for the existence of the corresponding spherical Goldberg polyhedra.

**Figure 5.**
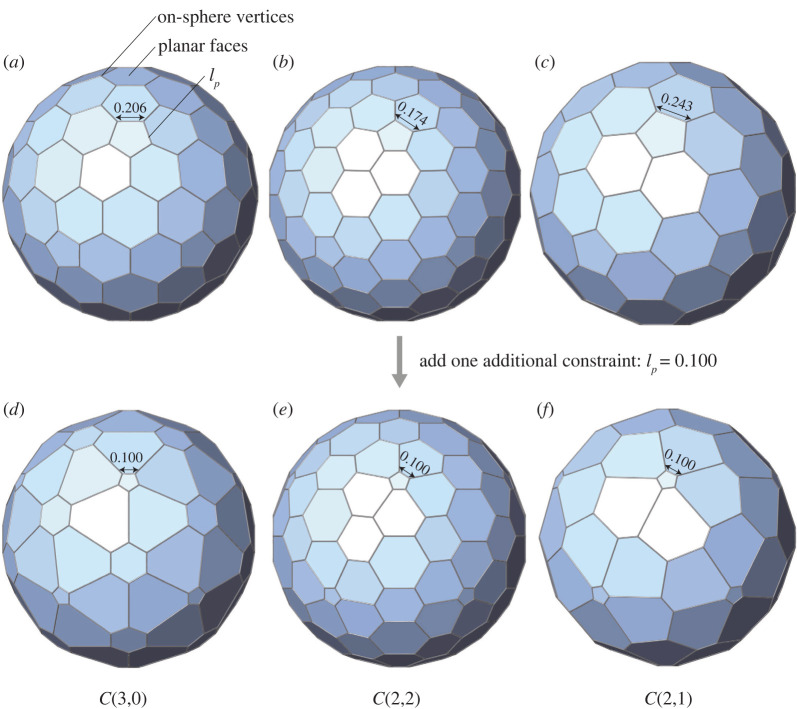
Spherical Goldberg polyhedra of different topology and geometry. (*a*) GP(3,0) with *l*_*p*_ = 0.206, (*b*) GP(2,2) with *l*_*p*_ = 0.174, (*c*) GP(2,1) with *l*_*p*_ = 0.243, (*d*) GP(3,0) with *l*_*p*_ = 0.100, (*e*) GP(2,2) with *l*_*p*_ = 0.100, (*f* ) GP(2,1) with *l*_*p*_ = 0.100.

We exemplarily test three cages, C(3,0), C(2,2) and C(2,1). The generated geometries are shown in figure 5*a*–*c*, with the edge length of regular pentagons equalling 0.206, 0.174 and 0.243, respectively. The corresponding data are detailed in table 1, cases 5–7, where all geometric errors have been successfully decreased below 10^−15^. Such nearly exact results suggest the possible existence of spherical Goldberg polyhedra GP(3,0), GP(2,2) and GP(2,1). For other pairs of (*m*, *n*), our method can be employed following the same process to investigate the existence of corresponding spherical Goldberg polyhedra and obtain nearly exact numerical models if available.

### 5.2. Case II: with one additional constraint on *l*_*p*_

The uniqueness of spherical Goldberg polyhedra for a specific topology is investigated by adding one additional constraint. If the second nearly exact solution (besides the primary nearly exact solution generated with no additional constraint) can be obtained, these results could serve as numerical evidence that the geometry of spherical Goldberg polyhedra corresponding to the specific topology is not unique.

By further requiring the pentagon’s edge length *l*_*p*_ to be equal to 0.100, the same cages, C(3,0), C(2,2) and C(2,1), are re-examined. The obtained geometries are shown in figure 5*d*–*f*, with corresponding data detailed in table 1, cases 8–10. It is clearly shown in table 1 that all errors are below 10^−15^. Therefore, for cages C(3,0), C(2,2) and C(2,1), two sets of different nearly exact solutions exist, which indicates that the corresponding spherical Goldberg polyhedra may be non-unique. For other pairs of (*m*,*n*), based on the numerical results, we conjecture that the solution of spherical Goldberg polyhedra may be non-unique; certain degrees of freedom may remain, which allow the geometry to be further adjusted.

### 5.3. Case III: with additional constraints on the number of different edge lengths

#### 5.3.1. Calculating the minimum *k*

For a spherical Goldberg polyhedra with certain degrees of freedom in its geometry, additional constraints may be applied to achieve other geometric properties that would bring extra practical benefits. In engineering and architecture, it is a long-lasting tradition to minimize the number of different edge lengths. This would allow mass production of struts, thereby significantly reducing the overall building fare.

To achieve this goal, a useful strategy is to integrate *k*-means clustering [28] and optimization. In clustering, given a target cluster number *k*, edges are partitioned into *k* clusters where each cluster includes edges of similar lengths. In optimization, each edge is adjusted towards the average length of its corresponding cluster by manipulating the positions of vertices. Every time the vertices are moved, a new round of clustering is performed to renew the clustering information for the next step of optimization. By iteratively performing the above process until convergence, edges in each cluster can be merged to be equal-length. Therefore, the number of different edge lengths are reduced to the number of clusters. However, *k* being too small would incur excessive constraints; in this case, edges in the same cluster would still possess different lengths in the final result.

To obtain the minimum *k* for the case of spherical Goldberg polyhedra, we propose an iteration framework as follows. We start by assigning *k* as *k*_0_ for the first iteration of clustering and optimization. Specifically, *k*_0_ equals the number of unique edges, and optimization aims to achieve planarity, inscribability and equilaterality (with each group). Then, based on the geometry generated from the previous iteration, we decrease *k* by one for the next iteration. By repeating this process until *k* reaches zero, the global iteration completes. Eventually, for each *k* ranging from 1 to *k*_0_, it corresponds to one cage with specific values of geometric errors. Therefore, the minimum *k* can be targeted according to the user-specified tolerance of the geometric errors.

#### 5.3.2. A numerical example

**Figure 6.**
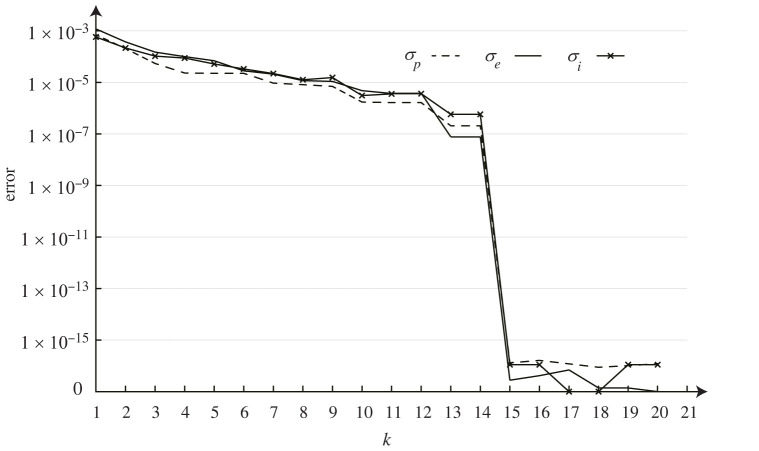
The plot of the relationship between errors and *k* for C(8,0).

**Figure 7.**
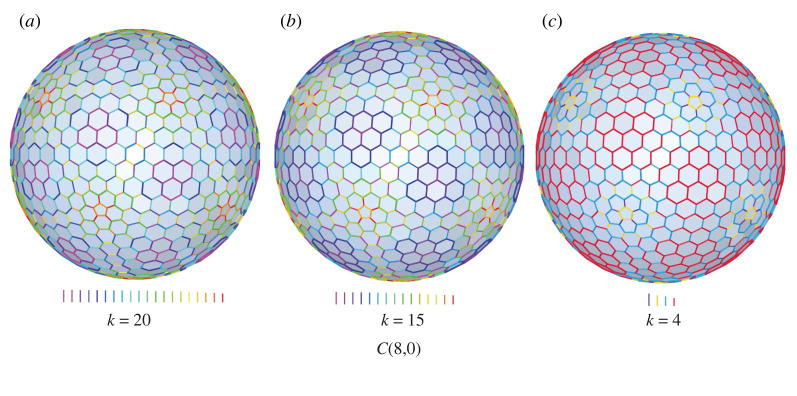
Spherical Goldberg polyhedra with different values of *k*. (*a*) *k* = 20, (*b*) *k* = 15, (*c*) *k* = 4. In each cage, edges of the same length are in the same colour.

A cage, C(8,0), is taken as an example to illustrate the effectiveness of the framework. The *k*-means function [29,30] in Matlab is adopted for the clustering. Starting with 20, which is the number of unique edges for C(8,0) as shown in figure 4*d*, *k* gradually decreases by one for each iteration until reaching zero. The initial spherical GP(8,0) with *k* = 20, is shown in figure 7*a*. Its corresponding geometric errors are listed in table 1, case 11. The overall relationship between the geometric errors and *k* is plotted in figure 6.

It is apparent in figure 6 that the errors increase as *k* reduces. Particularly, when *k* ≥ 15, all errors are lower than 10^−15^, meaning that nearly exact solutions can be obtained. Therefore, the minimum *k* for a nearly exact solution is 15. The corresponding geometry with *k* = 15 is shown in figure 7*b*, with the detailed data listed in table 1, case 12. Note that the runtime of case 12 is much higher than case 11, which is due to the inclusion of *k*-means clustering and the additional iterations required by the global framework.

For practical applications with errors ubiquitous and inevitable, considering the cost-effectiveness, a practical solution is often preferred compared with a nearly exact solution. A practical solution typically contains higher errors than the nearly exact solutions, yet still within a given tolerance, which is determined by the on-site circumstances, such as construction technique, building scale, material used, etc. For the spherical GP(8,0), if given a tolerance of 10^−4^ for all types of geometric errors, the minimum *k* can be significantly reduced to 4 according to the relationship plot (figure 6). The obtained geometry is shown in figure 7*c* with the corresponding geometric errors listed in table 1, case 13. In this case, only four types of edges are needed, which could remarkably diminish the overall building fare. Furthermore, if comparing it with the geometries of nearly exact solutions (figure 7*a*,*b*), one can hardly note any difference.

## 6. Conclusion

This study has presented the first framework that combines Goldberg’s method, symmetry analysis, parametrization and optimization technology to systematically determine the topology and control the geometry of Goldberg polyhedra. This method allows users to conveniently explore and generate cages with specified geometric properties, and thereby may serve as a powerful tool in diverse fields, including polyhedra in mathematics, dome design in architecture and engineering, fullerene in chemistry and virus capsids in biology. In detail, we first parameterize the cage’s geometry under the constraints of icosahedral symmetry and specified topology, and then manipulate the predefined independent variables through optimization to achieve a variety of desirable geometric properties. Various numerical examples have been conducted to demonstrate the effectiveness of the proposed method.

Furthermore, using the new method, we have successfully achieved nearly exact spherical Goldberg polyhedra with extremely low errors. The results presented in this study serve as strong numerical evidence for the existence of spherical Goldberg polyhedra, which may shed some light on these unusual geometries that have not yet been achieved or proved to exist. We also show that, with a given topology, the solution of spherical Goldberg polyhedra may not be unique, meaning that multiple different geometries may exist. Due to the remaining degrees of freedom, we further reduce the number of different edge lengths by performing *k*-means clustering and optimization iteratively. The obtained spherical Goldberg polyhedra with the minimum number of different edge lengths could significantly reduce the construction cost of spherical buildings. As extensions to this work, we will consider exploring other architecture-related geometric properties and adapting the new method to design freeform surfaces with periodic patterns in the future.

## Data Availability

The data are provided in electronic supplementary material [31].

## References

[1] Goldberg M. 1937 A class of multi-symmetric polyhedra. Tohoku Math. J., First Ser. **43**, 104-108.

[2] Caspar DL, Klug A. 1962 Physical principles in the construction of regular viruses. In *Cold Spring Harbor Symp. on Quantitative Biology*, vol. 27, pp. 1–24. Cold Spring Harbor, NY: Cold Spring Harbor Laboratory Press.

[3] Marks RW, Fuller RB. 1973 Dymaxion world of Buckminster fuller. New York, NY: Anchor Books.

[4] Gáspár O. 2021 Bauersfeld’s concept for the subdivision of the first built geodesic dome structure. In *Proc. of IASS Annual Symposia, Surrey*, vol. 2021, pp. 1–10. Madrid, Spain: International Association for Shell and Spatial Structures (IASS).

[5] Hart G. 2013 Goldberg polyhedra. In *Shaping space* (ed. M Senechal), pp. 125–138. New York, NY: Springer.

[6] Šiber A. 2020 Icosadeltahedral geometry of geodesic domes, fullerenes and viruses: a tutorial on the t-number. Symmetry **12**, 556. (10.3390/sym12040556)

[7] Tarnai T, Gáspár Z, Lengyel A. 2013 From spherical circle coverings to the roundest polyhedra. Phil. Mag. **93**, 3970-3982. (10.1080/14786435.2013.800652)

[8] Crick FH, Watson JD. 1956 Structure of small viruses. Nature **177**, 473-475. (10.1038/177473a0) 13309339

[9] Kroto HW, Heath JR, O’Brien SC, Curl RF, Smalley RE. 1985 C60: Buckminsterfullerene. Nature **318**, 162-163. (10.1038/318162a0)

[10] Fujita D, Ueda Y, Sato S, Mizuno N, Kumasaka T, Fujita M. 2016 Self-assembly of tetravalent Goldberg polyhedra from 144 small components. Nature **540**, 563-566. (10.1038/nature20771) 30905932

[11] Schwerdtfeger P, Wirz LN, Avery J. 2015 The topology of fullerenes. Wiley Interdiscip. Reviews: Comput. Mol. Sci. **5**, 96-145. (10.1002/wcms.1207) PMC4313690 25678935

[12] Brinkmann G, Goetschalckx P, Schein S. 2017 Comparing the constructions of Goldberg, Fuller, Caspar, Klug and Coxeter, and a general approach to local symmetry-preserving operations. Proc. R. Soc. A **473**, 20170267. (10.1098/rspa.2017.0267)

[13] Pottmann H, Eigensatz M, Vaxman A, Wallner J. 2015 Architectural geometry. Comput. Graph. **47**, 145-164. (10.1016/j.cag.2014.11.002)

[14] Pellis D, Wang H, Kilian M, Rist F, Pottmann H, Müller C. 2020 Principal symmetric meshes. ACM Trans. Graph. **39**, 127-1. (10.1145/3386569.3392446)

[15] Wang H, Pellis D, Rist F, Pottmann H, Müller C. 2019 Discrete geodesic parallel coordinates. ACM Trans. Graph. **38**, 1-13.(10.1145/3355089.3356541)

[16] Wang W, Liu Y, Yan D, Chan B, Ling R, Sun F. 2008 Hexagonal meshes with planar faces. *Dept. of CS, HKU, Tech. Rep*. See https://www.cs.hku.hk/data/techreps/document/TR-2008-13.pdf.

[17] Basso P, Del Grosso AE, Pugnale A, Sassone M. 2009 Computational morphogenesis in architecture: cost optimization of free-form grid shells. J. Int. Assoc. Shell Spatial Struct. **50**, 143-150.

[18] Lee T-U, Liu Y, Xie YM. 2022 Dividing a sphere hierarchically into a large number of spherical pentagons using equal area or equal length optimization. Comput.-Aided Des. **148**, 103259. (10.1016/j.cad.2022.103259)

[19] Rezaee Javan A, Lee T-U, Xie YM. 2022 Dividing a sphere into equal-area and/or equilateral spherical polygons. J. Comput. Des. Eng. **9**, 826-836. (10.1093/jcde/qwac031)

[20] Kitrick CJ. 2015 Equal edge hexagonal spherical tessellations. In *Proc. of IASS Annual Symposia, Amsterdam*, vol. 2015, pp. 1–12. Madrid, Spain: International Association for Shell and Spatial Structures (IASS).

[21] Schein S, Gayed JM. 2014 Fourth class of convex equilateral polyhedron with polyhedral symmetry related to fullerenes and viruses. Proc. Natl Acad. Sci. USA **111**, 2920-2925. (10.1073/pnas.1310939111) 24516137 PMC3939887

[22] Lengyel A, Gáspár Z, Tarnai T. 2017 The roundest polyhedra with symmetry constraints. Symmetry **9**, 41. (10.3390/sym9030041)

[23] Kitrick CJ. 2017 Icosahedral roundest polyhedra. In *Proc. of IASS Annual Symposia, Hamburg*, vol. 2017, pp. 1–10. Madrid, Spain: International Association for Shell and Spatial Structures (IASS).

[24] Dennis Jr JE, Gay DM, Walsh RE. 1981 An adaptive nonlinear least-squares algorithm. ACM Trans. Math. Softw. **7**, 348-368. (10.1145/355958.355965)

[25] Marquardt DW. 1963 An algorithm for least-squares estimation of nonlinear parameters. J. Soc. Ind. Appl. Math. **11**, 431-441. (10.1137/0111030)

[26] MATLAB. *9.8.0.1380330 (R2020a) Update 2*. Natick, MA: The MathWorks Inc. 2020.

[27] Moré JJ. 1978 The Levenberg-Marquardt algorithm: implementation and theory. In *Numerical analysis* (ed. GA Watson), pp. 105–116. Cham, Switzerland: Springer.

[28] Hartigan JA, Wong MA. 1979 Algorithm as 136: a *k*-means clustering algorithm. J. R. Stat. Soc. Ser. C (Applied Statistics) **28**, 100-108. (10.2307/2346830)

[29] Arthur D, Vassilvitskii S. 2006 k*-means++: the advantages of careful seeding*. Stanford InfoLab, CA, Technical Report 2006-13, June 2006.

[30] Lloyd S. 1982 Least squares quantization in PCM. IEEE Trans. Inf. Theory **28**, 129-137. (10.1109/TIT.1982.1056489)

[31] Liu Y, Lee TU, Rezaee Javan A, Xie YM. 2022 Extending Goldberg’s method to parameterise and control the geometry of Goldberg polyhedra. *Figshare*. (10.6084/m9.figshare.c.6125241) PMC9363989 35958093

